# Optimization of Deep Neural Networks Using SoCs with OpenCL

**DOI:** 10.3390/s18051384

**Published:** 2018-04-30

**Authors:** Rafael Gadea-Gironés, Ricardo Colom-Palero, Vicente Herrero-Bosch

**Affiliations:** Department of Electronic Engineering, Universitat Politècnica de València, Camino de Vera, s/n, 46022 València, Spain; rcolom@eln.upv.es (R.C.-P.); viherbos@eln.upv.es (V.H.-B.)

**Keywords:** evolutionary computation, embedded system, FPGA, deep neural networks, OpenCL, SoC

## Abstract

In the optimization of deep neural networks (DNNs) via evolutionary algorithms (EAs) and the implementation of the training necessary for the creation of the objective function, there is often a trade-off between efficiency and flexibility. Pure software solutions implemented on general-purpose processors tend to be slow because they do not take advantage of the inherent parallelism of these devices, whereas hardware realizations based on heterogeneous platforms (combining central processing units (CPUs), graphics processing units (GPUs) and/or field-programmable gate arrays (FPGAs)) are designed based on different solutions using methodologies supported by different languages and using very different implementation criteria. This paper first presents a study that demonstrates the need for a heterogeneous (CPU-GPU-FPGA) platform to accelerate the optimization of artificial neural networks (ANNs) using genetic algorithms. Second, the paper presents implementations of the calculations related to the individuals evaluated in such an algorithm on different (CPU- and FPGA-based) platforms, but with the same source files written in OpenCL. The implementation of individuals on remote, low-cost FPGA systems on a chip (SoCs) is found to enable the achievement of good efficiency in terms of performance per watt.

## 1. Introduction

Artificial neural networks (ANNs) are widely used in many areas of research and have produced very promising results.

However, the topological design of an ANN determines its usefulness, because it significantly influences the network’s performance [1]. When applying an ANN to a concrete problem, it is observed that a network that is too large tends to over-fit the training data, affecting its generalizability, whereas a network that is too small tends to encounter problems in learning from the training samples because of its limited representation capability. Furthermore, when good results are achieved, it is not clear whether those results are optimal among all network structures.

This uncertainty may be deemed acceptable by researchers if the other ANN conditions (for example, the numbers of training samples and input variables) are fixed; however, when all of these parameters must be evaluated as part of the same research, a long period of experimentation is required to determine the optimal topology. Curteanu and Cartwright [2] listed the main methods for obtaining the optimal topology: trial and error, empirical or statistical methods, hybrid methods, constructive and destructive algorithms and evolutionary strategies.

In structure design, evolutionary algorithms (EAs) may be employed in one of two ways: to evolve only the structure [3] or to simultaneously evolve both the structure and the connection weights [4].

Many researchers have focused on the simultaneous optimization of the network structure and the connection weights: Leung et al. [5] presented an improved genetic algorithm to simultaneously tune both the structure and the parameters, whereas Tsai et al. [6] used a hybrid Taguchi-genetic algorithm to tune both the network structure and the parameters. Ludermir et al. [7] presented an approach combining simulated annealing with tabu search for the simultaneous optimization of multilayer perceptron (MLP) network weights and architectures. Palmes et al. [8] used a mutation-based genetic neural network (MGNN) to replace backpropagation (BP) with a mutation strategy based on local adaptation of evolutionary programming (EP) to achieve weight learning. Niu et al. [9] applied improved particle swarm optimization using optimal foraging theory (PSOOFT) to train the free parameters (weights and bias) of an ANN and used a binary PSO algorithm to evolve the network architecture. Lu et al. [10] proposed a quantum-bit representation with which to codify a network, indicating not the actual links, but the probability of existence of the connections, thereby alleviating mapping problems and reducing the risk of discarding a potential candidate. Garro et al. [11] presented a methodology for automatically designing an ANN using three variants of particle swarm optimization algorithms (PSO, second generation PSO (SGPSO) and Nelder–Mead PSO (NMPSO)) to evolve the three principal components of an ANN: the set of synaptic weights, the connections or architecture and the transfer functions for each neuron. Young et al. [12] used a genetic algorithm in order to optimize the filter sizes and the number of filters in the convolutional layers. Their architectures consisted of three convolutional layers and one fully-connected layer.

With regard to the optimization of neural network architectures specifically needed for depth learning, the following works should be highlighted: Miikkulainen et al. [13] proposed a CoDeepNEAT-based Neuron Evolution of Augmenting Topologies (NEAT) to determine the type of each layer and its hyperparameters. Real et al. [14] used a genetic algorithm to design a complex CNN architecture through mutation operations and managing problems in filter sizes through zeroth order interpolation. Reinforcement learning, based on Q-learning [15], was used by Baker et al. to automatically generate high- performing CNN architectures for a given learning task. However, these promising results were only achieved with significant computational resources and a long execution time.

However, the use of EAs poses a major difficulty: the ability of an ANN to evolve into a superior ANN relies on the survival of the correct topology. To obtain this correct topology, it is necessary to ensure that individuals with the best topologies are the best ranked and that these individuals are retained as the best breeding individuals for the next generation. In the case of the simultaneous evolution of the structure and the connection weights, a good fitness value is not necessarily an accurate representation of the quality of the structure. In the event that a suitable fitness function is obtained, the most efficient way to perform the crossover of these individuals must be determined.

The other approach is to evolve only the structure and not the connection weights. The connection weights must then be learned after a near-optimal architecture is found. One major disadvantage of evolving the architecture without the connection weights is noisy fitness evaluations [16,17]. Different random initial weights may produce different training results. Therefore, structures with the same representation (genotype) may show quite different levels of fitness. This one-to-many mapping from the genotypes to the actual networks (phenotypes) may induce noisy fitness evaluations and result in misleading evolution. To reduce such noise, an architecture should usually be trained many times using different random initial weights. The average result is then used to estimate the genotype’s mean fitness. However, this method significantly increases the computation time required for fitness evaluation, which is one of the major drawbacks of using EAs. Therefore, the main motivation for our study is to demonstrate that our implementation permits the distribution of the computation across a heterogeneous network, efficiently decreasing the computational load.

The second objective of our study is to show that a neural network can be optimized to achieve good approximation performance not only on the training data, but also on unseen data for the same problem (generalization).

This paper is organized as follows: Section 2 describes the evolutionary optimization method. Section 3 describes the implemented platform. Experimental results are presented in Section 4. Conclusions are presented in Section 5.

## 2. Evolutionary Optimization Method

The objective of our evolutionary optimization method is to obtain the best-trained neural network to generate a solution to a problem with no apparent algorithmic solution.

The given conditions for which we seek to fulfill this objective are the following:

- We have a set of samples, and a number of clear targets for, the problem that we wish to solve (in general, classification, regression or pattern matching)
- For each sample, we have several inputs. There is no clear evidence indicating whether each of these inputs is necessary or important.
- Finally, we do not know whether all of the available samples are suitable for the training, validation or testing of our neural network.

Our method consists of the following phases:

Phase 1: Selection of the best inputs via evolutionary computation based on the delta test. When performing feature selection, it is important to avoid intervention from the neural network to ensure that the selection of the variables is independent of the network topology. Before conducting the current study, we investigated delta test optimization using genetic algorithms for regression problems with only one output [18]; however, this approach can also be extended to classification problems with multiple outputs. Because of the particular focus of this study, this phase of the methodology was not considered here. We are certain that different, more efficient methods could be used; however, in this experiment, our aim was to devise an orderly and efficient partitioning method for independent optimization and implementation.
Phase 2: Filtering of the samples through replicator neural networks [19].
Phase 3: Optimization of the neural network topology via heterogeneous evolutionary computation (this experimentation platform will be implemented and evaluated in this article 4.2).
Phase 4: Optimization of the initial neural network weights via evolutionary computation (this experimentation platform was implemented and evaluated in [20]).
Phase 5: Final training of a neural network with the topology obtained in Phase 3 and the initial weights obtained in Phase 4.

When using this method, it is very important to be able to ensure (and evaluate) the generalization capability of the optimal neural network obtained. To achieve this objective, we applied the following constraints:

- The set of samples after Phase 2 (filtering) was divided into four subsets: training, validation, optimization and testing. The training subset was used to train MLPs based on the fitness function of the evolutionary algorithm in Phases 3 and 4. The validation subset was used for early termination of the MLP training in Phases 3 and 4. The purpose of the optimization subset was to obtain the objective value(s) (for a single objective or multiple objectives) of the fitness function for each individual of the population. Finally, the test subset was used to evaluate the final optimized neural network. We followed the recommendation that the test subset should not be used for the identification of the best-performing trained neural network [21].
- In Phases 3 and 4, many MLP training runs were necessary. In our experiments developed for Phase 4 in [20], we used the resilient backpropagation algorithm. Now, in the essays proposed for Phase 3 that are presented in Section 4.2, the RMSpropalgorithm was selected for this purpose based on two considerations: the speed of the algorithm and, more importantly, the ease of hardware implementation for all technologies used in the heterogeneous platform. RMSprop is a very effective, but currently unpublished, adaptive learning rate method; however, it shares with many other algorithms (e.g., Adam [22] and Adadelta [23]) the same characteristics of the forward and backward phases that are of interest to us for acceleration through OpenCL. To improve the generalization properties of the RMSprop algorithm, we performed early termination on the validation subset.
- The final training run was performed using the Bayesian regularization algorithm [24] on the union of the training, validation and optimization subsets.
- In Phase 3 (topology optimization), we performed a multi-objective optimization in which the second objective of the evolutionary computation was to minimize the number of connections. This technique improved the generalizability of the resulting neural network [25].

### 2.1. Optimization of the Topology

Phase 3 represents the key feature of our method: the separation of the feature selection process (Phase 1) and the identification of the initial weights (Phase 4) from the topology optimization. It is very common to address all three of these goals simultaneously in optimization via evolutionary computation [26]; however, this choice impairs the topology optimization performance.

As noted in the Introduction, the separation of the optimization of the network topology and the initialization of the weights is challenging.

In our first attempt to separate the optimization problem, we performed several initializations of the weights for each individual in the population (each with a different topology) and calculated the fitness function for the genetic algorithm based on the average performance among these initializations.

The three main problems with this approach are as follows:

- Enormous computational effort is required. Our work and further details on the computational effort are presented in Section 3.
- Control of random number generation, which is necessary for the initialization of the weights during training, is difficult. Without such control, the best individuals in the population may be lost because a good individual can suffer decreases in their fitness function value in subsequent generations of the evolutionary algorithm (see Section 2.1.1).
- It is difficult to determine the best method for extracting the best individuals when different initializations are averaged (see Section 2.1.2).

#### 2.1.1. Control of Random Number Generation

The second problem was easily identified because the best individual varied from generation to generation; this behavior was observed independent of the method used to extract the best individual (third problem).

In our method, it is important to obtain the fitness function values of the best individuals, which may decrease during evolution in the genetic algorithm. To achieve this objective, the random generator streams of the individuals must be controlled, without affecting the random generation properties of the main genetic algorithm, while preventing the same random number generator from being used for different individuals. All of these properties were achieved by applying the following procedures in the fitness function:

- Definition of a random generator stream with a unique seed for each individual: Therefore, three variables were necessary for the evolutionary computations related to the neural network topology: the number of neurons in the first hidden layer, the number of neurons in the second hidden layer and the seed of the random generator stream.
- Resetting the number generator streams to reproduce the results for the best individuals.
- Utilization of different sub-streams with different weight initializations for each individual.

#### 2.1.2. Weight Initialization

**Figure 1:**
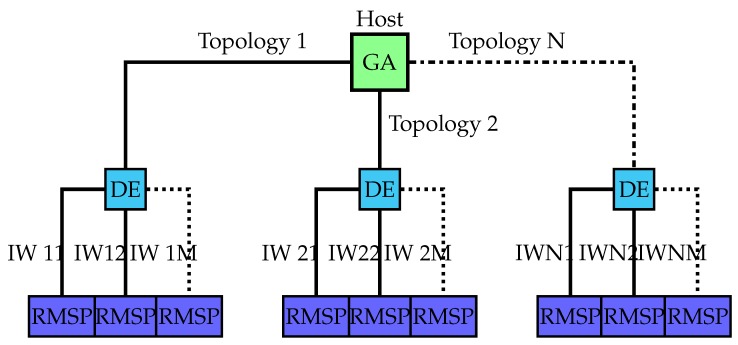
Structure of Phase 3 for GATOPODE. DE, differential evolution.

To address the third problem, three alternative approaches were tested:

- A fitness function with the goal of minimizing the mean squared error (MSE) or the mean of the MSE when training the same topology with a given number of executions of the RMSprop algorithm, each beginning with a different weight initialization. This alternative is called GATOPOMINor GATOPOMEAN.
- A fitness function with the goal of obtaining the best fitness function value for the evolutionary computation of the initial weights (as in Phase 4); in other words, the execution of a subordinate evolutionary computation for each individual as part of the main evolutionary computation. This alternative is called GATOPOGA or GATOPODE, depending on the evolutionary algorithm used in the second-level evolutionary computation: a genetic algorithm (GA) or differential evolution (DE) (see Figure 1). This scheme is obtained based on the studies carried out by [27].
- A fitness function with the goal of minimizing the MSE of resilient BP when the initial weights have been established using auto-encoders [28]. This technique is very commonly used for the non-supervised training of deep neural networks (DNNs). This alternative is called GATOPODNN.

A detailed assessment of these three alternatives is outside the scope of this study. However, the computational effort required for each alternative is obvious, as is the need for high flexibility in the implementation of the individuals for the genetic algorithm.

If we suppose that a population of 100 individuals is considered in the main genetic algorithm, we can assess the number of RMSprop training runs needed in one generation as follows:

- With 50 different initializations, we would need 5000 RMSprop training runs in each generation.
- With 500 individuals considered in the second-level evolutionary computation (GA or DE), we would need 50,000 RMSprop training runs in each generation of the main genetic algorithm.
- With an MLP with three layers of weights, we would need 300 RMSprop training runs for two layers of MLP weights in each generation and 100 RMSprop training runs for three layers of MLP weights in each generation.

## 3. Heterogeneous Computational Platform

In our implementation, the neural network optimization method introduced in Section 2 (specifically, Phase 3 of the method) requires a host processor responsible for the main genetic algorithm, which manages the derivation of the ANN topology.

The number of variables to be handled is only three, and therefore, the size of the population to be managed is rather limited. For each individual, a neural network must be trained that consists of two hidden layers with one set of initial weights, in the case of the non-supervised method (GATOPODNN); various random weight initializations (GATOPOMEAN and GATOPOMIN); or initial weights obtained via a nested genetic algorithm, in the most complex case (GATOPOGAand GATOPODE).

**Figure 2:**
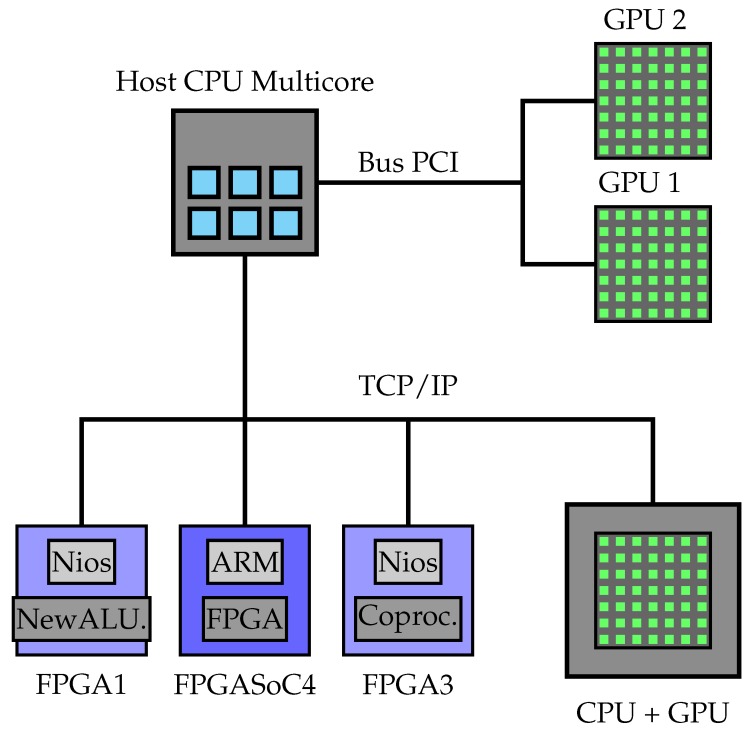
Heterogeneous computational platform.

To implement the host processor, we opted for a generalist solution based on a multicore CPU using Python, enabling us to easily adapt the solution to other platforms to generate experimental samples, perform quick implementation comparisons with existing toolboxes for global optimization methods and neural network implementations and obtain a straightforward representation of the results. This generalist host platform had access to a GPU via a graphics board connected to the main board. Our study does not focus on this host processor, as it is a very common platform, but the minimum requirements are described. The structure can be seen at the top of Figure 2.

Our main modification to this platform was to add the ability for the host processor to connect (via simple socket clients) to a set of embedded processors (soft or hard) using FPGA technology, thereby providing us with many different system-on-a-chip (SoC) solutions (Figure 2). This modification enabled us to adopt the following alternative implementations of the individuals for the main genetic algorithm:

- local individuals
  - CPU cores
  - GPU processors
- remote individuals on FPGAs
  - FPGA0 soft embedded processor
  - FPGA1 soft embedded processor + soft embedded processor with neural instructions
  - FPGA2 soft embedded processor + programmable coprocessor in software (technological solution provided in [20])
  - FPGA3 soft embedded processor + reconfigurable coprocessor
- remote individuals on FPGA-SoCs
  - FPGA-SoC0 hard embedded processor
  - FPGA-SoC1 hard embedded processor + soft embedded processor with neural instructions
  - FPGA-SoC2 hard embedded processor + programmable coprocessor in software
  - FPGA-SoC3 hard embedded processor + reconfigurable coprocessor (technological solution provided in this paper work)
- remote individuals with OpenCL solutions
  - FPGA-SoC4 hard embedded processor + reconfigurable kernel
  - FPGA4 soft embedded processor + reconfigurable kernel
  - CPU1 + PCI Express GPU kernel
  - CPU2 + PCI Express FPGA kernel

We started with solutions [20] that were technologically possible based on devices using the Altera Cyclone IV family (remote individuals on FPGAs of type FPGA2) in which the soft processor is ALTERA’s NIOS2 microprocessor. It was found that the FPGA-technology-enabled solutions (remote individuals on FPGA-SoCs) required the ability to draw on the features provided by devices in the Intel FPGA Cyclone V family, which are common in new devices from other FPGA vendors and that are essential for understanding the main differences from our previous work from an implementation technology point of view:

- Embedded hard cores (Integrated ARM Cortex-A9 MPCore Processor System)
- Runtime reconfiguration
- Partial reconfiguration
- Use of OpenCL solutions and, therefore, quasi-compatible solutions, with others technologies (CPU-GPU)

There are several precedents for the implementation of neural networks on FPGAs. The work in [29] developed a coprocessor for convolutional neural networks (CNNs) and investigated the acceleration it achieved when applied before other technologies (CPU). The work in [30] presented a training platform for a reconfigurable topology. The drawback to this application is that it must re-synthesize the topology each time it is changed and then implement the new topology on the FPGA. Another previous application involved the implementation of a fixed-topology ANN and a BP algorithm for training [31]. The work in [32] proposed a BP algorithm implementation on a software-reconfigurable FPGA. Pinjare et al. [33] developed an FPGA accelerator for online training. The work in [34] evaluated their methodology using an FPGA. They were also able to extract the best performance density from the FPGA, although they were the only researchers to date to employ floating-point numeric representations in their computations, and the soft embedded processor was used only to assist with CNN accelerator startup, communication with the host CPU and time measurement. Their accelerator achieved a 4.79× performance gain over a 16-threaded CNN implementation on a baseline CPU, with 5.1× less power consumption and a 24.6× energy reduction.

In the majority of these studies, the implementations of ANNs on FPGAs (with online training) have relied on very complex and efficient PEs; however, these implementations present great difficulties in terms of communication, flexibility and, above all, their adaptability to technological changes. New work such as [35] may represent a change in this sense, in which this lack of flexibility can be overcome; but in general terms, few works are capable of delivering better performance results than GPUs, unless we move on to those recognizable as binarized neural networks that have a drastic reduction in the resolution of computations performed [36] or by comparison with SoC GPU [37].

The first step in the implementation of individuals on our platform was to resolve the issue of communication with the host for the particular type of algorithm running on the host. Resolving this issue requires that a portion of the platform that is handling the individuals is used to implement a processor for managing a socket server. Therefore, in each of our FPGA-based solutions, there is one main processor (soft or hard macro) responsible for this task, usually requiring an operating system to assist in socket management.

To address the issue of platform flexibility, we first focused on implementations that allow the implementation of neural networks with different topologies using an architecture acting as a coprocessor that acquires instructions from the main processor. Therefore, a new task was assigned to the main processor: compilation of the instructions for the coprocessor. However, we are certain that in the future, accelerators with finer granularity will be realized on FPGAs (called kernels), which will allow direct compilation based on OpenCL and will be able to be reused in various parts of the execution of the original algorithm while permitting run-time reconfiguration.

Finally, the issue of ensuring both flexibility and adaptability was resolved by using a design flow based on an OpenCL language that allowed us to reconfigure and compare different technologies for enhancing lossless application efficiency in the implementation methodology. The use of these languages prepared for a multi-platform compilation currently constitutes one of the lines of greatest production in the implementation of deep neural networks associated with CNN networks [38,39,40].

### 3.1. Implementation of the Individuals

The implementation of the individuals in Phase 3 is based on the calculation of the fitness function for the population considered in the main genetic algorithm; therefore, the main computational effort is focused on training one ANN via RMSprop BP (RBP). However, we must more finely tune the tasks involved to enable the calculation of individuals on a remote board:

1. Sending the training set for the application from the host of the main genetic algorithm to the remote board calculating the individuals. This information must be sent to the remote board only once. If the number of generations of the main genetic algorithm is sufficiently large, the time required for communication to the remote node is usually negligible. The time spent on this task is denoted by $T_{dt}$.
2. Transmitting the variables necessary for the evolutionary computation of the neural network topology: the number of neurons in the first hidden layer, the number of neurons in the second hidden layer and the seed of the random generator stream. This information must be sent for each new individual created by the remote board. The time spent on this task is denoted by $T_{dc}$.
3. Sending the command from the host to the remote board to initiate the calculation of the fitness function for an individual. The time spent on this task is denoted by $T_{sc}$.
4. Performing a large number of training iterations ($N_{t}$) for an ANN via RBP. The time required for this training can be decomposed into a fixed initialization time for the algorithm ($T_{ini}$) and the time required for some number of iterations (*E*) of a loop, commonly referred to as epochs; within each iteration of this loop, there is another set of iterations determined by the number of mini-batches into which the training samples have been divided ($N_{m}$), and each mini-batch iteration is broken down into two main phases: the calculation of the partial derivative of the error with respect to each weight and the updating of the weights. The amounts of time spent on each of these phases are denoted by $T_{cw}$ and $T_{uw}$, respectively. After training, we must calculate the fitness values for the test set ($T_{test}$).
5. Sending the response for each individual from the remote board to the host to signal the end of the fitness function calculation and communicate the calculated value. The time spent on this task is denoted by $T_{rr}$.

Finally, we have the following time for the execution of *G* generations of the main genetic algorithm with a population of *P* individuals when we use one board for the implementation of the individuals:

(1) $$T_{dt}+G\cdot P\cdot \left(T_{dc}+N_{t}\cdot \left(RBP\right)+T_{rr}\right)$$

being:

(2) $$RBP=T_{ini}+E\cdot N_{m}\cdot \left(T_{cw}+T_{uw}\right)+T_{test}$$

The remaining time required for optimization via the genetic algorithm is the computation time for the main processor in our platform ($T_{main}$), which is independent of the type of implementation (local or remote) that is used for the individuals.

**Figure 3:**
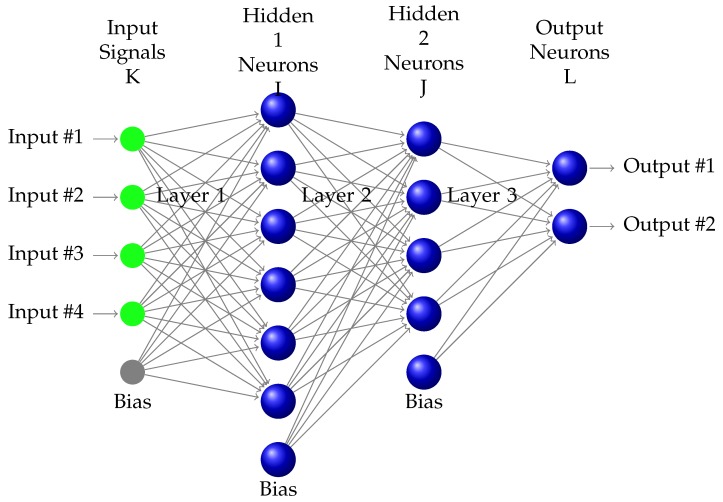
Multiplayer perceptron.

The following subsections will consider Equation (2). We consider an MLP with three layers: two hidden layers (*I* and *J*) and an output layer (*L*) (i.e., 4-6-4-2 in Figure 3).

#### 3.1.1. Calculation of Weight Changes $T_{cw}$

The calculation of the partial derivative of the error with respect to each weight is usually separated into three phases, which are performed iteratively for each pattern. If there are $N_{p}$ patterns in one mini-batch, then the time required for the process can be expressed as follows:

(3) $$T_{cw}=N_{p}\cdot \left(T_{f}+T_{b1}+T_{b2}\right)$$

The three steps involved in this calculation are performed for one pattern *m* at a time, where the network has *L* layers of neurons and $N_{l}$ neurons in each layer. The partial derivative of the error with respect to each weight is calculated as follows:

- $T_{f}$: Forward step. Apply the pattern $y_{i}^{K}$ to the input layer and propagate the signal forward through the network until the final outputs $y_{i}^{L}$ have been calculated for each *i* (the neuron index) and *l* (the layer index).
  (4) $$\begin{array}{c} u_{i}^{l}=\sum_{j=0}^{N_{l-1}}w_{ij}^{l}y_{j}^{l-1}\; \end{array}$$
  (5) $$\begin{array}{c} y_{i}^{l}=f\left(u_{i}^{l}\right) \end{array}$$
  (6) $$\begin{array}{c} y_{i}^{\prime l}=f^{\prime }\left(u_{i}^{l}\right)\\ 1\leq i\leq N_{l},\;1\leq l\leq L \end{array}$$
  where *y* is an activation, *w* is a weight and *f* is a non-linear function.
- $T_{b1}$: Backward Step 1. Compute the δ values for the output layer *L* and for the preceding layers by propagating the errors backwards using:
  (7) $$\begin{array}{c} \delta_{j}^{L}=f^{\prime }\left(u_{j}^{L}\right)\left(t_{i}-y_{i}\right) \end{array}$$
  (8) $$\begin{array}{c} \theta_{i}^{l-1}=\sum_{i=1}^{N_{l}}w_{ij}\delta_{i}^{l} \end{array}$$
  (9) $$\begin{array}{c} \delta_{j}^{l-1}=f^{\prime }\left(u_{j}^{l-1}\right)\theta_{i}^{l-1}\\ 1\leq i\leq N_{l},\;2\leq l\leq L \end{array}$$
  where δ is the error term, *t* is the target and $f^{\prime }$ is the derivative of the function *f*.
- $T_{b2}$: Backward Step 2. When we obtain the delta errors in Backward Step 1, we can simultaneously obtain the accumulated partial derivative of the error with respect to each weight as follows:
  (10) $$\begin{array}{c} e=1/2\sum_{i=1}^{N_{l}}{\left(t_{i}-y_{i}^{L}\right)}^{2} \end{array}$$
  (11) $$\begin{array}{c} {}^{m}\frac{\partial e}{\partial w_{ij}^{l}}=\delta_{i}^{l}y_{j}^{l-1} \end{array}$$
  (12) $$\begin{array}{c} {}^{m}cw_{ij}^{l}={}^{m-1}cw_{ij}^{l}+{}^{m}\frac{\partial e}{\partial w_{ij}^{l}}\\ 1\leq i\leq N_{l},\;1\leq l\leq L \end{array}$$
  where cw is the accumulated direction of the error gradient. Once we have finished processing all of the patterns, the accumulated result will be:
  (13) $$\begin{array}{c} cw_{ij}^{l}=\frac{\partial E}{\partial w_{ij}^{l}} \end{array}$$
  (14) $$\begin{array}{c} E=\frac{1}{2N_{p}}\sum_{m=1}^{N_{p}}\sum_{i=1}^{N_{l}}{\left(t_{i}-y_{i}^{L}\right)}^{2}\\ \;1\leq l\leq L \end{array}$$

All of the elements needed in Backward Step 2 are provided at the same time as the elements necessary for Backward Step 1. Therefore, it is possible to perform these two final steps simultaneously during the execution of each layer. This action reduces the number of steps to two: a forward step and a backward step. However, in the OpenCL implementation, it is necessary to clearly differentiate these steps and only begin Backward Step 2 when Backward Step 1 has completely finished.

#### 3.1.2. Update of Weights $T_{uw}$

The updating of the ANN weights requires the realization of all of the weights of the ANN when each epoch has finished.

(15) $${w_{ij}}^{(t+1)}={w_{ji}}^{\left(t\right)}+\Delta {w_{ij}}^{\left(t\right)}$$

(16) $$\Delta {w_{ij}}^{\left(t\right)}={\left(\frac{\partial E}{\partial w_{ij}}\right)}^{\left(t\right)}\cdot \frac{\Delta_{ij}^{\left(t\right)}}{\sqrt{r_{ij}^{\left(t\right)}}}$$

(17) $$r_{ij}^{\left(t\right)}=\left(1-\gamma \right)\cdot {\left({\left(\frac{\partial E}{\partial w_{ij}}\right)}^{2}\right)}^{\left(t\right)}+\gamma \cdot r_{ij}^{\left(t-1\right)}$$

(18) $${\Delta_{ij}}^{\left(t\right)}=\left\{\begin{array}{c} \begin{array}{cc} \left(1+\eta^{+}\right){\cdot \Delta_{ij}}^{\left(t-1\right)}, & \mathrm{if}{\left(\frac{\partial E}{\partial w_{ij}}\right)}^{\left(t-1\right)}*{\left(\frac{\partial E}{\partial w_{ij}}\right)}^{\left(t\right)}>0\\ \left(1-\eta^{-}\right){\cdot \Delta_{ij}}^{\left(t-1\right)}, & \mathrm{if}{\left(\frac{\partial E}{\partial w_{ij}}\right)}^{\left(t-1\right)}*{\left(\frac{\partial E}{\partial w_{ij}}\right)}^{\left(t\right)}<0\\ {\Delta_{ij}}^{\left(t-1\right)}, & \mathrm{else} \end{array}\\ where\;0<\eta^{-}<1<\eta^{+} \end{array}\right.$$

Once the equations necessary for the execution of a neuronal network training in an individual (what we have called RBP) have been broken down, it is necessary to look at the following Algorithm 1.

In this algorithm, we place in each execution line:

- to the right, the equations involved as listed above and
- to the left, an identification that allows us to understand which of these operations are implemented by our OpenCL kernel in the three main versions developed and that now we will detail.

As we advance in the three versions, we will observe how our kernel will assume more lines of execution of our algorithm, which will result in a greater complexity of the OpenCL developed in exchange for a clear decrease in the data communications between the main microprocessor (ARM) and the kernel (FPGA) in each individual.

### 3.2. Version 1: Algorithm with Kernel of Type Matrix Multiplication

To implement Algorithm 1 in OpenCL, our main candidates for the kernels are Lines 1, 2, 3, 4, 5, 6, 7 and 8 of Algorithm 1. Each of these kernels would have a similar structure based on matrix-matrix multiplication and on the path dependence that exists between them. Therefore, a better solution would be to implement a single kernel and reuse it eight times in each iteration. With this approach, we would use the same kernel 8·E times in our algorithm.

Equation (19) shows the matrix-matrix multiplications necessary for the forward phase. Equation (20) shows the matrix-matrix multiplications necessary for Backward Phase 1. Equation (21) shows the matrix-matrix multiplications necessary for Backward Phase 2. In each matrix-matrix multiplication, the size of the matrices must be modified, and thus, our OpenCL implementation must permit the modification of these parameters. Note that it is necessary to transfer new input matrices and read new matrix output via memory buffers every time the kernel is reused.

**Algorithm 1:** RMSprop backpropagation.
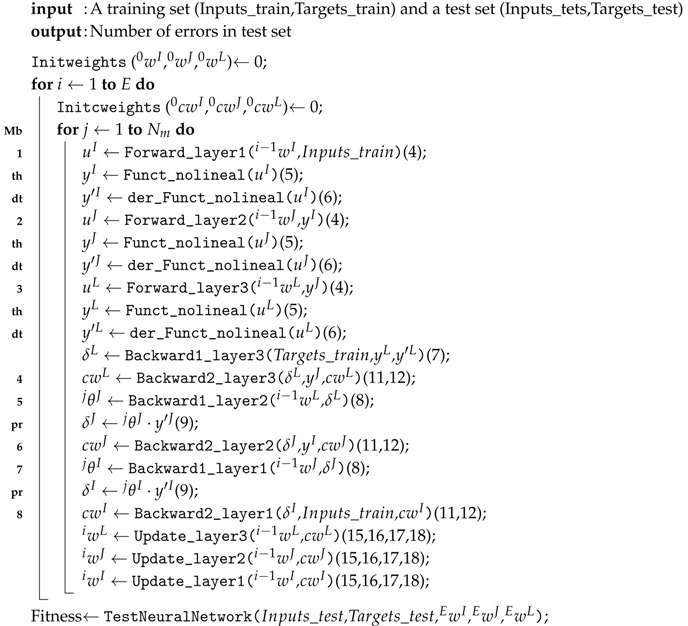


To illustrate the operation of our OpenCL kernel, we can select any of the matrix multiplications in Equations (19)–(21) and proceed to group the elements of each matrix into the sub-blocks Aand B with dimensions of b×b (typically called tiles), where *b* is the work-group size of the OpenCL kernel.

(19) $$\left\{\begin{array}{c} \left(\begin{array}{ccc} I_{11} & \ldots & I_{1n}\\ \vdots & \ddots & \vdots \\ I_{m1} & \cdots & I_{mn} \end{array}\right)\left(\begin{array}{ccc} w_{11} & \ldots & w_{k1}\\ \vdots & \ddots & \vdots \\ w_{1n} & \cdots & w_{kn} \end{array}\right)=\left(\begin{array}{ccc} u_{11} & \ldots & u_{1k}\\ \vdots & \ddots & \vdots \\ u_{m1} & \cdots & u_{mk} \end{array}\right)\to \left\{\begin{array}{c} m=numPattern\\ n=numInputs\\ k=numHidden1 \end{array}\right\}\to forward-layer1\\ \left(\begin{array}{ccc} y_{11} & \ldots & y_{1n}\\ \vdots & \ddots & \vdots \\ y_{m1} & \cdots & y_{mn} \end{array}\right)\left(\begin{array}{ccc} w_{11} & \ldots & w_{k1}\\ \vdots & \ddots & \vdots \\ w_{1n} & \cdots & w_{kn} \end{array}\right)=\left(\begin{array}{ccc} u_{11} & \ldots & u_{1k}\\ \vdots & \ddots & \vdots \\ u_{m1} & \cdots & u_{mk} \end{array}\right)\to \left\{\begin{array}{c} m=numPattern\\ n=numHidden1\\ k=numHidden2 \end{array}\right\}\to forward-layer2\\ \left(\begin{array}{ccc} y_{11} & \ldots & y_{1n}\\ \vdots & \ddots & \vdots \\ y_{m1} & \cdots & y_{mn} \end{array}\right)\left(\begin{array}{ccc} w_{11} & \ldots & w_{k1}\\ \vdots & \ddots & \vdots \\ w_{1n} & \cdots & w_{kn} \end{array}\right)=\left(\begin{array}{ccc} u_{11} & \ldots & u_{1k}\\ \vdots & \ddots & \vdots \\ u_{m1} & \cdots & u_{mk} \end{array}\right)\to \left\{\begin{array}{c} m=numPattern\\ n=numHidden2\\ k=nuOutputs \end{array}\right\}\to forward-layer3 \end{array}\right.$$

(20) $$\left\{\begin{array}{c} \left(\begin{array}{ccc} \delta_{11} & \ldots & \delta_{1n}\\ \vdots & \ddots & \vdots \\ \delta_{m1} & \cdots & \delta_{mn} \end{array}\right)\left(\begin{array}{ccc} w_{11} & \ldots & w_{1k}\\ \vdots & \ddots & \vdots \\ w_{n1} & \cdots & w_{nk} \end{array}\right)=\left(\begin{array}{ccc} \theta_{11} & \ldots & \theta_{1k}\\ \vdots & \ddots & \vdots \\ \theta_{m1} & \cdots & \theta_{mk} \end{array}\right)\to \left.\left\{\begin{array}{c} m=numPattern\\ n=numOutputs\\ k=numHidden2 \end{array}\right.\right\}\to backward1-layer2\\ \left(\begin{array}{ccc} \delta_{11} & \ldots & \delta_{1n}\\ \vdots & \ddots & \vdots \\ \delta_{m1} & \cdots & \delta_{mn} \end{array}\right)\left(\begin{array}{ccc} w_{11} & \ldots & w_{1k}\\ \vdots & \ddots & \vdots \\ w_{n1} & \cdots & w_{nk} \end{array}\right)=\left(\begin{array}{ccc} \theta_{11} & \ldots & \theta_{1k}\\ \vdots & \ddots & \vdots \\ \theta_{m1} & \cdots & \theta_{mk} \end{array}\right)\to \left.\left\{\begin{array}{c} m=numPattern\\ n=numHidden2\\ k=numHidden1 \end{array}\right.\right\}\to backward1-layer1 \end{array}\right.$$

(21) $$\left\{\begin{array}{c} \left(\begin{array}{ccc} \delta_{11} & \ldots & \delta_{1n}\\ \vdots & \ddots & \vdots \\ \delta_{m1} & \cdots & \delta_{mn} \end{array}\right)\left(\begin{array}{ccc} y_{11} & \ldots & y_{1k}\\ \vdots & \ddots & \vdots \\ y_{n1} & \cdots & y_{nk} \end{array}\right)=\left(\begin{array}{ccc} cw_{11} & \ldots & cw_{1k}\\ \vdots & \ddots & \vdots \\ cw_{m1} & \cdots & cw_{mk} \end{array}\right)\to \left.\left\{\begin{array}{c} m=numOutputs\\ n=numPattern\\ k=numHidden2 \end{array}\right.\right\}\to backward2-layer3\\ \left(\begin{array}{ccc} \delta_{11} & \ldots & \delta_{1n}\\ \vdots & \ddots & \vdots \\ \delta_{m1} & \cdots & \delta_{mn} \end{array}\right)\left(\begin{array}{ccc} y_{11} & \ldots & y_{1k}\\ \vdots & \ddots & \vdots \\ y_{n1} & \cdots & y_{nk} \end{array}\right)=\left(\begin{array}{ccc} cw_{11} & \ldots & cw_{1k}\\ \vdots & \ddots & \vdots \\ cw_{m1} & \cdots & cw_{mk} \end{array}\right)\to \left.\left\{\begin{array}{c} m=numHidden2\\ n=numPattern\\ k=numHidden1 \end{array}\right.\right\}\to backward2-layer2\\ \left(\begin{array}{ccc} \delta_{11} & \ldots & \delta_{1n}\\ \vdots & \ddots & \vdots \\ \delta_{m1} & \cdots & \delta_{mn} \end{array}\right)\left(\begin{array}{ccc} y_{11} & \ldots & y_{1k}\\ \vdots & \ddots & \vdots \\ y_{n1} & \cdots & y_{nk} \end{array}\right)=\left(\begin{array}{ccc} cw_{11} & \ldots & cw_{1k}\\ \vdots & \ddots & \vdots \\ cw_{m1} & \cdots & cw_{mk} \end{array}\right)\to \left.\left\{\begin{array}{c} m=numHidden1\\ n=numPattern\\ k=numInputs \end{array}\right.\right\}\to backward2-layer1 \end{array}\right.$$

OpenCL requires that the number of work-groups is chosen such that the size of the NDRangeindex space is evenly divided in each dimension. Therefore, it is necessary for our BP algorithm to guarantee this property of the matrix dimensions in Equations (19)–(21).

**Figure 4:**
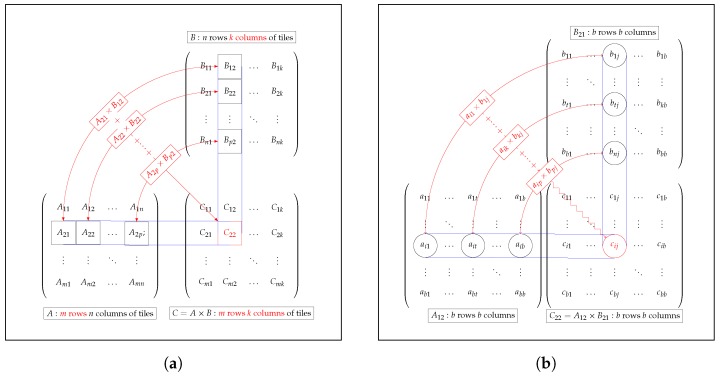
Structure of matrix multiplication. (**a**) Matrix-matrix multiplication; (**b**) tile-tile multiplication.

To compute a sub-block $C_{22}$ of C (see Figure 4a) with dimensions of b×b, we need the corresponding *b* rows of A and the corresponding *b* columns of B. Now, if we also divide these *b* rows of *A* and *b* columns of *B* into sub-blocks $A_{sub}$ and $B_{sub}$ with dimensions of b×b, we can iteratively update the values in $C_{22}$ by summing the results of multiplying $A_{sub}\times B_{sub}$.

If we take a closer look at the computation of a single element (in Figure 4b), we see that there is a great deal of data reuse within a tile. For example, all elements in row *i* of tile $C_{22}$ are computed using the data from row *i* of tile $A_{12}$, and all elements in column *j* of tile $C_{22}$ are computed using the data from column *j* of tile $A_{12}$; this phenomenon is common to all tile-tile multiplications.

Let us translate this abstract image into actual OpenCL code. The technique here is to share the data from tiles $A_{sub}$ and $A_{sub}$ within a work-group via local memory. To maximize the benefits of data reuse, we might attempt to make these tiles as large as possible.

In our experiments, we used 2D work-groups with dimensions of 4 × 4 (or b×b), 8 × 8 and 16 × 16. The $C_{sub}$ tiles were also of the same dimensions.

### 3.3. Version 2: Introduction of Non-Linear Function in the Kernel

Our experience in designing accelerators for the BP algorithm ([41]) indicates that one of the most effective means of achieving acceleration is usually to replace the software implementation of the hyperbolic tangent with a hardware implementation.

When working with OpenCL, we consider introducing functions labeled with the word “th” (see Algorithm 1) in the preceding kernel implementations (Lines 1, 2, and 3). Therefore, the kernel must support non-linear hyperbolic tangent operations in calls to the kernel belonging to the “forward” phase of the BP algorithm.

If we introduce the hyperbolic tangent in our OpenCL file, the definition of *tanh(x)* is *sinh(x)/cosh(x)*, which is equivalent to the three formulae given in Equation (22) below.

(22) $$\left\{\begin{array}{c} \left(e^{x}-e^{-x}\right)/\left(e^{x}+e^{-x}\right)\\ (1-\left(2/\left(e^{2x}+1\right)\right)\\ \left(e^{2x}-1\right)/\left(e^{2x}+1\right) \end{array}\right.$$

The results for this type of solution show that the cost of FPGA hardware resources is high, and the benefits achieved are not decisive (see the results for raw tanh OpenCL in Table 1).

**Figure 5:**
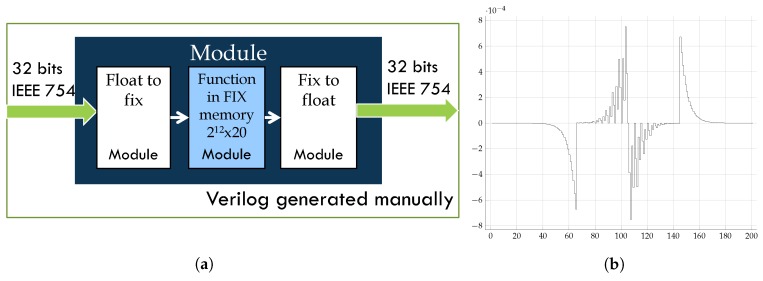
Implementation of tanh. (**a**) Structure of RTL tanh implementation; (**b**) error of the RTL tanh implementation.

Therefore, we have made efforts to insert our own Verilog RTL code in place of the current standard implementation of the hyperbolic tangent function in OpenCL (see Figure 5a). In the case of Version 16 of the Altera SDK for OpenCL, such a procedure is available to all users, and one can create an OpenCL library in either OpenCL or RTL; however, in earlier versions, the process is more complicated.

Our results for the resources used, the speed performance and the power consumption are shown in Table 1 in the row labeled RTL tanh IP. These results are nearly optimal for the majority of the parameters. The decrease in the maximum clock frequency for the kernel is the only worsening of a parameter observed with our implementation; however, we believe that with a latency of five, we could improve this performance. In Section 4, we will demonstrate the efficiency achieved in terms of the execution speed of the BP algorithm using this RTL solution.

Figure 5b shows the error of our implementation of the hyperbolic tangent compared with the software implementation of the same function. It is clear that this error is precisely the price we must pay for this approach and will only be acceptable if the general procedure for obtaining the optimal neural network topology is not impaired by this requested resolution. We know from experience with the isolated algorithm that this resolution is sufficient; however, the impact of this approach on the evolutionary optimization method can only be determined once the platform is complete.

### 3.4. Version 3: Introduction of the Derivative of the Tangent and Internal Storage of Variables in the Kernel

The last modification to the kernel is to also implement the computations included in the “dt” and “pr” lines of Algorithm 1. This modification requires us to also have a proper implementation of the derivative of the hyperbolic tangent (based on look-up tables (LUTs) for tanh). With the internal storage of the variables obtained by the kernel in the forward phase and Backward Phase 1, these variables can be reused in Backward Phase 2, thereby avoiding many of the transfers of information between the host and the kernel that would otherwise be necessary throughout the successive reuses of the kernel. This forces the kernel to have eight different operating modes. Another consequence of this modification to the kernel is that its size increases.

## 4. Performance Evaluation

### 4.1. ARM Versus ARM + FPGA Kernel

#### 4.1.1. Preliminary Comments

We optimized the data processing efficiency of the three versions of our kernel by implementing strategies such as unrolling loops, setting work-group sizes and specifying compute units and work-items.

According to the available Intel FPGA guides, there are generally two ways to increase the extent of parallelism:

1. Use single instruction, multiple data (SIMD) techniques for vectorized data load/store. We use num_simd_work_items and reqd_work_group_size as kernel attributes; multiple work-items from the same work-group will run at the same time. In all versions, with regard to the parameterization kernel, we consider the following attributes:
  - reqd_work_group_size: four types of two-dimensional work-groups (4 × 4, 8 × 8, 16 × 16 and 32× 32), which correspond to the size of the sub-matrices (tiles) into which each of the arrays is divided (see Figure 4b).
  - num_simd_work_items: three values (1, 2 and 4).
2. Replicate the compute resources on the remote device (FPGA). We use num_compute_units as a kernel attribute; multiple work-groups will run at the same time.

By applying these two techniques, we ensured that there would be multiple work-groups, each with multiple work-items, running simultaneously on the FPGA device.

For each of these kernel solutions, we varied the size of the matrices involved in Algorithm 1. To modify the matrix size, we can vary five variables; four are related to the network topology, and the fifth is related to the size of the mini-batches used for training. This last variable, the mini-batch size, is the most influential because it is involved in all of the matrix multiplication algorithms. The topology variables are described as follows:

- numInputs: This variable depends on the type of problem to be solved using the neural network. It is a parameter that will not be changed by our evolutionary system throughout its entire execution. It influences two of our eight matrix multiplications (see Equations (19)–(21)).
- numOutputs: This variable also depends on the type of problem to be solved using the neural network and is a parameter that will not be changed by our evolutionary system throughout its entire execution. It affects three of our eight matrix multiplications (see Equations (19)–(21)).
- numHidden1: This variable shows no dependence on the type of problem to be solved. It is a design parameter and is therefore subject to optimization by our evolutionary algorithm. It affects five of our eight matrix multiplications (see Equations (19)–(21)).
- numHidden2: This variable also shows no dependence on the type of problem to be solved; it is also a design parameter and therefore subject to optimization by our evolutionary algorithm. It affects six of our eight matrix multiplications (see Equations (19)–(21)).

Based on this analysis, we opted to maintain our study settings for four of the five variables in each test, and we individually varied our hardware solutions (mentioned above), the number of neurons in the first hidden layer, the number of neurons in the second hidden layer and the number of patterns per mini-batch.

In each test, only one variable was changed, and the remaining values were set as follows: numPatterns: 65,536 numInputs: 78 numHidden1: 256 numHidden2: 256 numOutputs: 10.

#### 4.1.2. Analysis of Hardware Results

For each version of the kernel, our criterion was to select the FPGA implementation that yielded the best acceleration performance. In the Table 2, we present the optimal parameters used for each version of the kernel and the hardware resources used in their implementations.

#### 4.1.3. Analysis of Performance Results

**Figure 6:**
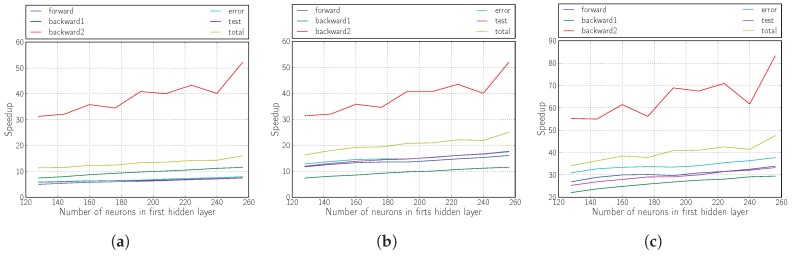
Speedup of the kernel when varying the number of neurons in the first hidden layer. (**a**) Version 1; (**b**) Version 2; (**c**) Version 3.

**Figure 7:**
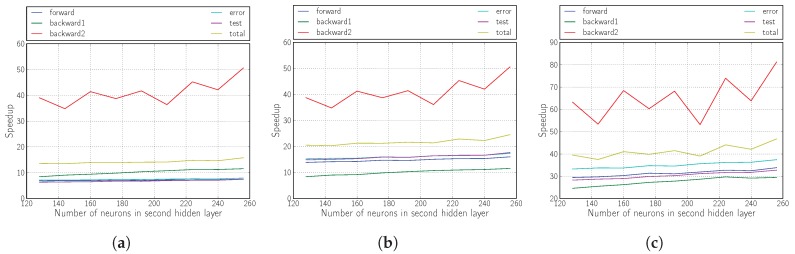
Speedup of the kernel when varying the number of neurons in the second hidden layer. (**a**) Version 1; (**b**) Version 2; (**c**) Version 3.

**Figure 8:**
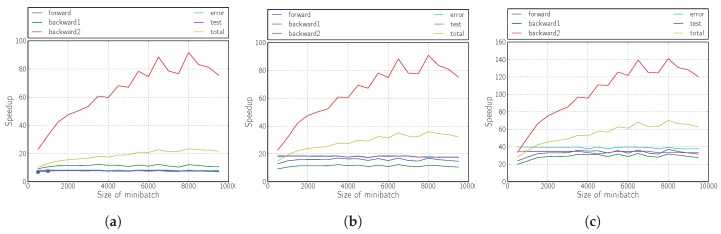
Speedup of kernel when varying when varying the mini-batch size. (**a**) Version 1; (**b**) Version 2; (**c**) Version 3.

The results shown in Figure 6, Figure 7 and Figure 8 illustrate that the proposed solution for accelerating the RMSprop algorithm using OpenCL is truly effective compared with its execution on the ARM Cortex-A9 processor.

The achieved performances correspond to an acceleration of more than 40, whether we modify the sizes of the hidden layers (the topology) or the mini-batch size.

It is also demonstrated that the inclusion of logic in the kernel (Versions 2 and 3) yields fully satisfactory results, tripling the yield compared with the version that only performs matrix multiplication (Version 1).

The improvement provided by Version 2 is mainly evidenced by the improvement of the forward phase due to the calculation of the non-linear functions required within the kernel in the FPGA.

For Version 3 and as we have already mentioned, the improvement in performance comes from the partial improvement of all the phases of the algorithm, but above all from the drastic decrease in the number of data transfers between the host and the kernel.

Needless to say, this comparison with the ARM Cortex-A9 processor alone is not sufficient proof that the heterogeneous platform proposed in this article is adequate. For this reason, in the following subsection, we report a performance evaluation of a prototype of the proposed platform.

### 4.2. Phase 3 Heterogeneous Platform

#### 4.2.1. Preliminary Comments

In our first test, we compared two platforms: a completely homogeneous platform, with only a four-core i7 processor (for all tasks: host and individuals) or only a 12-core Xeon processor, and a heterogeneous platform, with an i7 or Xeon processor and four DE1-SoC FPGA boards. The main characteristics of these boards are:

- FPGA device:
  - Cyclone V SoC 5CSEMA5F31C6 Device
  - Dual-core ARM Cortex-A9 (HPS)
  - 85 K programmable logic elements
  - 4450 Kbits embedded memory
  - 6 fractional PLLs
  - 2 hard memory controllers.
- Memory device:
  - 64-MB (32 M × 16) SDRAM on FPGA
  - 1-GB (2 × 256 M × 16) DDR3 SDRAM on HPS and shared with FPGA. OpenCL kernels access this shared physical memory through direct connection to the HPS DDR hard memory controller.

These platforms were used to execute Phase 3 of our evolutionary optimization method for the topology optimization of a two-layer neural network, with a mini-batch size of 8000 samples, using a genetic algorithm considering a population of four individuals and executed through five generations.

To limit the experiment, we opted for multi-objective optimization (minimization of the mean quadratic error for the set of test samples and minimization of the number of connections).

**Figure 9:**
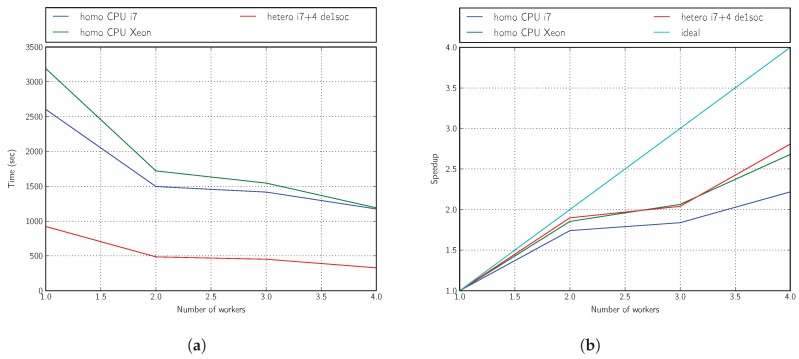
Performance: five generations, four individuals. (**a**) Time; (**b**) speedup.

From the results shown in Figure 9a,b, we can conclude that the heterogeneity introduced by using remote embedded systems is advantageous for acceleration. On the abscissa axis in these figures, we vary the number of workers. For the homogeneous platform, this means increasing the size of the processor pool; for the heterogeneous platform, it means adding more FPGA boards.

Our accelerated deployment using the ARM + OpenCL kernel (based on Intel FPGA Cyclone V technology) is nearly three-times faster than that using only one of the four cores available on an Intel(R) Core(TM) i7 860 CPU @2.80 GHz or one of the 12 cores available on an Intel(R) Xeon(R) E5-2620 ver2 CPU @ 2.10 GHz, and the acceleration performance can be slightly further improved by progressively including a larger number of workers, i.e., using more cores on the homogeneous platform or more boards on the heterogeneous platform (up to 10 boards).

#### 4.2.2. Performance Efficiency

In our second test, we extended the utilization of the heterogeneity of the platform to the implementation of individuals, for which one also can use exclusively the cores of the host machine or can include the use of remote FPGA boards.

In addition, four maximum optimization platforms have been included in our comparisons: two homogeneous platform (i7 and Xeon platforms) that attempted to take full advantage of the multicore processor by means of optimization for better performance on CPUs (a CPU backend using neon 1.8.1 by Intel Nervana) and two heterogeneous platforms with GPUs: one that took advantage of the acceleration provided by GeForce GTX 670 GPUs and high-performance libraries (a GPU backend using neon 1.8.1 by Intel Nervana) and the other that took advantage of the acceleration provided by GeForce GTX Titan GPUs and high-performance libraries (a GPU backend using neon 2.1 by Intel Nervana).

**Figure 10:**
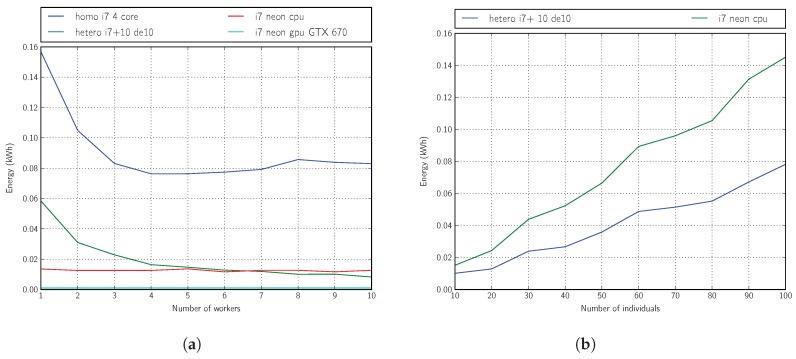
Energy consumption: three generations for the i7 platform. (**a**) Fixed number of individuals: 16; (**b**) fixed number of workers: 10.

Finally, we present in Figure 10a,b the energy efficiency information obtained from each of these platforms (related to i7 platform) for the realization of three generations in Phase 3 of our optimization method with a mini-batch size of 8000 samples.

**Figure 11:**
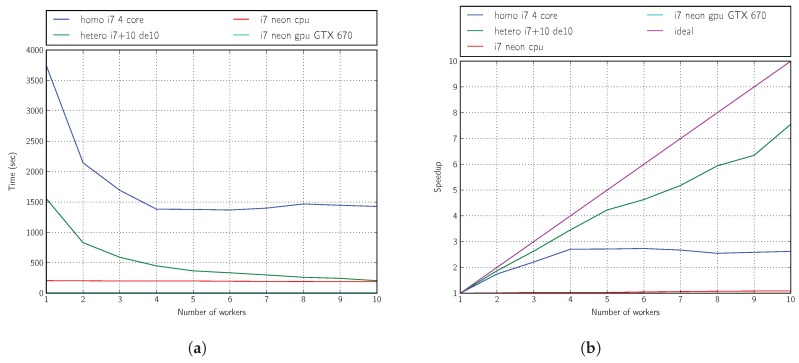
Performance: three generations, 16 individuals, i7 platform. (**a**) Time; (**b**) speedup.

**Figure 12:**
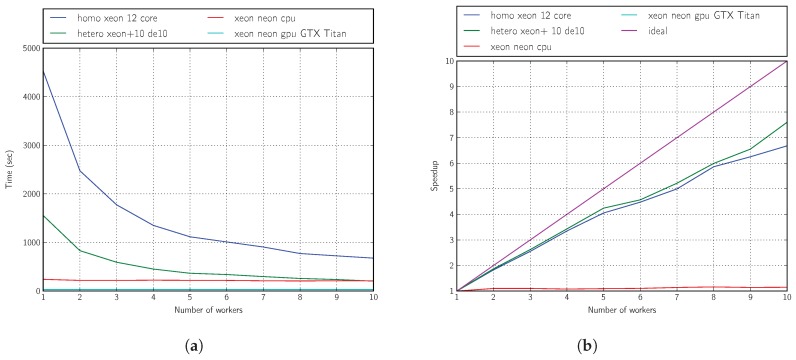
Performance: three generations, 16 individuals, Xeon platform. (**a**) Time; (**b**) speedup.

From the results shown in Figure 11a,b and Figure 12a,b, we can conclude that SoC boards can make an interesting contribution to improving the performance of multicore machines.

In Figure 10a, we can see, by means of the variation in the number of workers of the heterogeneous platform (or the same thing by varying the number of FPGA plates), that when we reach 10 SoC boards, we can already improve the energy efficiency of an entire multicore processor completely dedicated to the execution of the optimization algorithm.

If we apply this topology of platform with 10 SoC boards and now perform an optimization experiment by varying the number of individuals of the main genetic algorithm, we can see in Figure 10b the best energy efficiency of the heterogeneous versus homogeneous platform.

Another, very different situation that we may consider for comparison is the use of a host with a general-purpose GPU (GPGPU) device when there is a very-high-efficiency kernel available for the algorithm of interest. The number of SoC boards required to match the performance in such a case may be very high.

## 5. Conclusions

In this article, we have reported the development of an efficient and flexible approach that allows us to perform highly complex computations using a heterogeneous platform. We have shown that OpenCL, in combination with FPGAs, is an appropriate tool for this purpose. We have seen how an optimization algorithm can be used to train a neural network and that part of the process requires the optimization of the neural network topology; our results show that using OpenCL, one can easily increase the optimization speed by a factor on the order of 3N/C for network topologies of limited size, where N is the number of Socs used and C is the number of cores of the homogeneous multicore platform to be accelerated. In terms of energy efficiency, an improvement by a factor of approximately 5N/C can be achieved by implementing the calculations for the individuals considered in the genetic algorithm using low-cost SoC boards (<100 dollars). Nevertheless, when compared with highly optimized GPGPU implementations, the SoC approach still has a long way to go; although it is possible to achieve the same performance with a dozen low-cost boards, a GPGPU is the more cost-effective solution. Notably, the technologies (multi-CPU-FPGA and GPGPU) used here are not the latest-generation versions, which include the Intel Math Kernel Library (MKL) for multi-CPU operations, the Cyclone 10 family of FPGAs and the Kepler architecture with the cuBLASlibrary for GPUs; however, we do not believe that adopting these technologies would change the final assessment. It is important to note that two types of FPGA + OpenCL-based accelerator platforms exist: those in which the host processor is embedded in the same device as the FPGA (which we call Socs) and those in which the host processor is external to the device, where the FPGA is located on the accelerator board and communication is achieved through external means, most commonly PCIe and Ethernet ports.

Our intent is to incorporate FPGA + OpenCL solutions of this type into our platform, replacing the current low-cost boards with boards of much higher capacity and performance, using technologies based on Arria 10. In our future experiments, we intend to compare these solutions with GPGPU-based OpenCL solutions to obtain a much more coherent test bench based on which we can draw better substantiated conclusions. We will also be able to perform more detailed assessments of the flexibility and speed that can be achieved using both technologies and the potential effects of working with more limited resolutions, which, in some cases, is the only way to outperform GPGPUs.

## Figures and Tables

**Table 1:** Comparison between tanh implementations.

	ΔLUTs 4–5	ΔRegisters	ΔMemory	ΔDSP	ΔLatency	ΔPower	Frequency
	Variables		Blocks 10 kbits	Blocks	Cycles	Dissipation mW	MHz
OpenCL tanh	4511	9127	49	25	45	202.86	136.67
RTL IPtanh	161	163	10	0	4	18.98	133.07

**Table 2:** Hardware resources of kernel FPGA.

	Version 1	Version 2	Version 3
**reqd_work_group_size**	(16,16,1)	(16,16,1)	(16,16,1)
**num_simd_work_items**	4	4	1
**nnum_compute_units**	1	1	2
**Logic utilization**	22,995/32,070	23,500/32,070	22,293/32,070
**(in ALMs)**	72%	84%	70%
**Total registers**	51,357	52,259	52,011
**Total block**	1,323,804/4,065,280	1,653,928/4,065,280	1,770,586/4,065,280
**memory bits**	33%	41%	44%
**Total DSP**	72/87	72/87	48/87
**Blocks**	83%	83%	55%
**HPSPower**	1392.92 mW	1392.92 mW	1392.92 mW
**FPGA and HPS Power**	2524.70 mW	2959.60 mW	3147.25 mW
